# Editorial: Improvement of melanoma immune checkpoint blockade therapy with potential combinatorial regiments

**DOI:** 10.3389/fimmu.2022.1065937

**Published:** 2022-10-31

**Authors:** Xueyan Wang, Xiaowei Liu, Willy Hugo, Lu Si, Hubing Shi

**Affiliations:** ^1^ Laboratory of Integrative Medicine, Clinical Research Center for Breast, State Key Laboratory of Biotherapy, West China Hospital, Sichuan University, Chengdu, Sichuan, China; ^2^ Division of Dermatology, Department of Medicine, David Geffen School of Medicine, University of California, Los Angeles, CA, United States; ^3^ Key Laboratory of Carcinogenesis and Translational Research (Ministry of Education), Department of Renal Cancer and Melanoma, Peking University Cancer Hospital and Institute, Beijing, China

**Keywords:** immune checkpoint blockade (ICB), melanoma, immune checkpoint inhibitors (ICIs), biomarker, immune-related adverse events (irAEs), combination therapy

Melanoma is regarded as one of the most aggressive types of skin cancer. Immune checkpoint blockade (ICB), specifically blockade of programmed cell death protein 1 (PD-1), programmed death ligand 1 (PD-L1), or cytotoxic T-lymphocyte-associated antigen 4 (CTLA-4), has emerged as a pivotal clinical treatment for patients with advanced or metastatic cutaneous melanoma (1). Although patients who received ICB therapy achieved durable tumor remission and control, not all subtypes of melanoma patients benefited from this treatment (2, 3). Illustration of this mechanism of non-response to ICB therapy will improve the long-term outcomes of patients with melanoma. Furthermore, the extensive application of ICB drugs (also referred to as immune checkpoint inhibitors (ICIs)) is usually accompanied by drug resistance and immune-related adverse events (irAEs), which greatly hinder the benefits of patients from ICB therapy (4, 5). Therefore, novel biomarkers, targets, or combination therapies are urgent to determine patients who respond to ICB therapy, improve the therapeutic effects of ICB drugs, and avoid irAEs in melanoma. All the above issues are highlighted in this Research Topic Improvement of Melanoma Immune Checkpoint Blockade Therapy with Potential Combinatorial Regiments and shortly introduced in the Editorial. This collection aims to comprehensively summarize the latest progress and strategies for enhancing the therapeutic efficacy of ICB drugs in treating melanoma and provide new biomarkers for predicting the response of melanoma patients to ICB treatment.

As mentioned above, different subtypes of melanomas respond differently to ICIs. Acral and mucosal melanomas, two predominant subtypes in Asian countries including China, respond poorly to current therapies including immunotherapies (6–9). To elucidate this difference, Mao et al. provided a comprehensive review of the unique features of acral and mucosal melanomas. They believed that differences in clinicopathological characteristics, mutational landscapes, and tumor immune microenvironment may result in disparate outcomes of melanoma treatment. Then, they also discussed that combination therapy with ICIs may contribute to improving the clinical outcomes of these two subtypes. Similarly, Liu et al. found that the genomic subtype melanoma with *CCND1* amplification also responds less well to ICIs. They concluded that *CCND1* amplification resulted in immunosuppressive of the tumor microenvironment (TME), down-regulation of angiogenesis, and activation of metabolism signals, indicating that CDK4/6 inhibitors alone or combined with ICIs and inhibiting metabolism pathways may be effective for this subtype. Furthermore, the response of individual patients to anti-PD-1 monotherapy is also highly heterogeneous. To distinguish patients with different responses to anti-PD-1 monotherapy, Bai et al. established risk-scoring models for progression-free survival (PFS) and overall survival (OS), based on demographic characteristics and routinely tested results from two clinical trials. The results showed that neutrophil/lymphocyte ratio, derived neutrophil-lymphocyte ratio, and lactate dehydrogenase were all negatively associated with poor prognosis, which provided classification criteria for patients in different risk subcategories.

Additionally, biomarkers of response to ICIs have attracted increasing attention in melanoma research. Tumoral mutation burden is a classical biomarker for predicting the efficacy of ICB treatment (10, 11). This theory is further confirmed in melanoma by Gorgun et al. In their study, cisplatin-induced mutagenic DNA damage increased tumoral mutational loads. The combination of cisplatin and ICB drugs inhibited tumor growth *in vitro* and vivo, which broadens the way for the application of ICB therapy. Moreover, Yan et al. identified nine genes, including CCL5, GZMA, GBP5, GZMH, LAG3, IRF1, PRF1, NKG7, and PSMB10, promoted CD8+ T cell infiltration and are all related to prognosis by analyzing mRNA data from tumor and para-cancerous tissue. They claimed that these signatures could serve as biomarkers to assess response to immunotherapy for melanoma. Interestingly, ICIs generally work *via* T-lymphocytes, but recent studies showed that B cells might be involved in regulating the efficacy of ICIs (12, 13). Nevertheless, Wulfken et al. presented a case of one patient with metastatic melanoma and revealed that B cells were not needed for the efficacy of ICIs in this patient. Therefore, this issue should be further studied.

Although a majority of patients experienced durable tumor regression after receiving ICIs, drug resistance eventually led to tumor recurrence after initial remission (14). The development of resistance may be caused by insufficient immune responses, such as the lack of tumor antigens and immunosuppressive of TME (15). Therapeutic cancer vaccines (TCVs) are considered to be an emerging field that enhances the curative effect of ICIs by increasing immune response against tumors (16). Therefore, Ellingsen et al. discussed the potential of telomerase as a promising target for TCVs and provided optimized schemes, such as in combination with ICIs, for enhancing its therapeutic effect. Notably, with the wide application of ICIs, irAEs, such as colitis, rash, and autoimmune pneumonitis, have emerged endlessly, which have become important issues to be solved in this therapy (17). Bai et al. demonstrated that anti-PD-1 monotherapy elicited colitis in melanoma and induced CD8+ effector T cells to infiltrate into colitis lesions. This process involved the activation of PI3K/AKT/mTOR pathway in CD8+ effector T cells at lesions. Co-targeting PD-1 and mTOR with anti-PD-1 monoclonal antibody and sirolimus effectively inhibited tumor growth and relieved anti-PD-1 monoclonal antibody-induced colitis, which provided a new therapeutic strategy for the treatment of irAEs in clinics.

Taken together, although ICB therapy has achieved remarkable success in treating melanoma, the development of novel targets, biomarkers, and combinations remain required to improve its efficacy. We hope that this collection of the Research Topic will be enlightening for those devoted to improving the therapeutic effect of ICB therapy in melanoma or other cancers.

## Author contributions

HS supervised and conceived, and designed the project. XW reviewed all article of this research topic and wrote the original manuscript. XL reviewed and re-edited the manuscript. WH and SL reviewed and improved the manuscript. All authors approved the final paper.

## Conflict of interest

The authors declare that the research was conducted in the absence of any commercial or financial relationships that could be construed as a potential conflict of interest.

## Publisher’s note

All claims expressed in this article are solely those of the authors and do not necessarily represent those of their affiliated organizations, or those of the publisher, the editors and the reviewers. Any product that may be evaluated in this article, or claim that may be made by its manufacturer, is not guaranteed or endorsed by the publisher.
